# Physiotherapy for Multiple Sclerosis Patients From Early to Transition Phase: A Scoping Review

**DOI:** 10.7759/cureus.30779

**Published:** 2022-10-27

**Authors:** Vaishnavi Warutkar, Purva Gulrandhe, Shwetambari Morghade, Rakesh Krishna Kovela, Moh'd Irshad Qureshi

**Affiliations:** 1 Department of Physiotherapy, Ravi Nair Physiotherapy College, Datta Meghe Institute of Medical Sciences, Wardha, IND; 2 Department of Physiotherapy, Nitte Institute of Physiotherapy NITTE (Deemed to be University), Mangaluru, IND

**Keywords:** early care, transition phase, home program, covid-19, rehabilitation, multiple sclerosis

## Abstract

Multiple sclerosis (MS) is a chronic neurological disease that has an impact when they are at the most susceptible aspects of personal, professional, and social development. MS affects about 2.2 million individuals worldwide, with the majority of individuals experiencing relapses regularly. The progression of the disease's complex nature, the challenges in selecting the appropriate intervention, and a multitude of symptoms necessitate a systematic approach to the individual that includes both pharmacology and neurorehabilitation. Kinesiotherapy, exercise rehabilitation, massage, and hydrotherapy are all forms of physiotherapy that are used as part of rehabilitation. Physical exercise will mitigate the potential effects of akinesia and so enhance the functioning capacities of all bodily systems, regardless of the severity of the illness. An early examination by a physical therapist who is familiar with MS is advised to develop a customized training and/or lifestyle physical exercise program. Although hospital-based rehabilitation programs appear to have a higher impact, numerous studies have found that home-based rehabilitation is helpful. The constraint imposed by COVID-19 has an influence on the well-being of persons with multiple sclerosis. People with MS would be particularly affected, since they might be considered an at-risk group for serious COVID-19 in a variety of ways, and MS health-related data requirements increased significantly.

## Introduction and background

Multiple sclerosis (MS) is a chronic neurological condition that has an impact on young adults when they are at the most susceptible aspects of personal, professional, and social development [1]. It is an autoimmune inflammatory condition that has an impact on the central nervous system (CNS) [2]. The damage to the CNS causes a variety of symptoms, including alterations in cognitive function [3]. In Multiple Sclerosis, the nerve fibers in the encephalitis and spinal cord showed demyelination. This neurological condition produces unpredictability in motor, sensory, and cognitive functions, as well as mental and physical disorders [4]. Multiple sclerosis affects about 2.2 million individuals worldwide, with the majority of individuals experiencing relapses regularly. The frequency of relapses in the initial periods of the condition has been linked to impairment progression, with a larger percentage of remission resulting in a faster progression of disabilities. Apart from severe neurological relapses, relatively extensive neurodegeneration begins initially in the disease course, resulting in disability progression. As a result, an early decrease in relapse rate and neurodegeneration are regarded as critical in restricting the progression of the condition’s consequences [5,6]. The progression of the disease's complex nature, the challenges in selecting the appropriate intervention, and a multitude of symptoms necessitate a systematic approach to the individual that includes both pharmacology and neurorehabilitation [7]. Effective treatment methods to help reduce disorder activity and impairment advancement are still needed. Amongst the most effective non-pharmacological (additional) therapeutic interventions was exercise training. For individuals with Multiple Sclerosis, exercise is safe, acceptable, and has a low risk of side effects [5]. Kinesiotherapy, exercise rehabilitation, massage, and hydrotherapy are all forms of physiotherapy that are used as a component of rehabilitation. Physiotherapy in multiple sclerosis aims to improve the range of motion through compensatory mechanisms that involve the stimulation of effector capabilities and behavior, resulting in the patient restoring functioning rather than movement. Physical exercise will mitigate the potential effects of akinesia and so enhance the functioning capacities of all bodily systems, regardless of the severity of the illness. Physical activity helps not only the physical ability of individuals with multiple sclerosis but also their mood and attitude toward the exercises [7]. We have to learn more regarding specialized training physiotherapists need to give the proper type of assistance since it may need them to function differently to help people self-manage their physical work [8].

## Review

Method

The methodology for this scoping review was guided by the framework presented by Arksey and O'Malley (2005), with comments from Levac, Colquhoun, and O'Brien (2010). Scoping reviews offer a thorough examination of the articles. Scoping reviews look for published scientific proof and notions that support a particular study topic. For this study, a scoping review was chosen to acquire an overview of the academic literature on the role of physiotherapy at various phases of multiple sclerosis. Because this scoping review can give an overview of the available literature on various approaches to physical therapy for Multiple Sclerosis and various elements of MS.

Data sources and searches

An electronic search was done in Scopus, Embase, Medline, CINAHL, Pedro, PubMed, and Google Scholar from 2010 to August 2021. These sources were chosen for their comprehensive coverage of related healthcare fields, including physiotherapy. To ensure that all essential research was collected, systematic reference list checks and citation monitoring of retrieved papers using Google Scholar were carried out. Multiple sclerosis, early physiotherapy, the transition phase, and home program were utilized as search terms.

Study selection

For this scoping review to capture the breadth of various topics on Multiple Sclerosis articles, included were those who address physical therapy management in the early and late phases of the disease, different physiotherapy approaches for different symptoms of MS transition of environment for the patient from hospital to home, home program, and rehabilitation of MS patient in COVID-19 pandemic.

Charting extraction

A form for extracting data was made. To organize the data, authors, publication year, country of origin, publication kind or source, methodology, a conceptual technique including terminology used, terminology description, and rational implementation, including theoretical basis and context, were utilized.

Data synthesis and analysis

**Figure 1:**
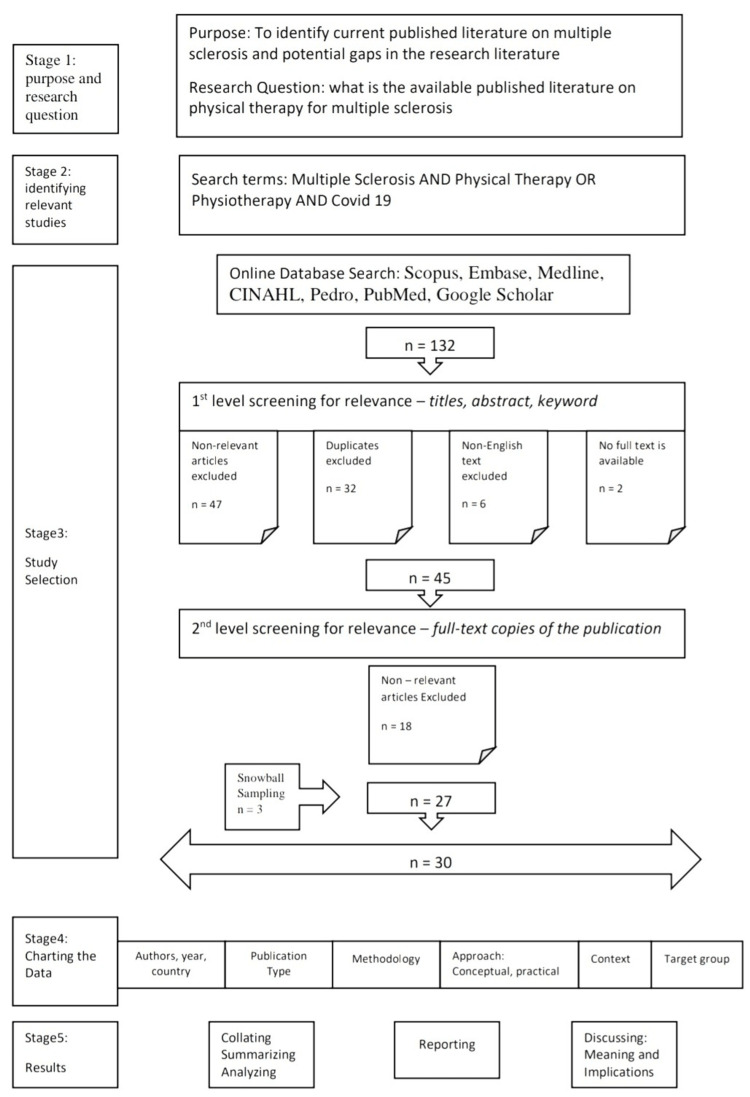
PRISMA flow chart on the available published literature on Physical Therapy for Multiple Sclerosis

After data charting, information was organized by publication year, country of origin, sources or categories of publications, and methodology used. The occurrence of these groups was assessed to identify the most common intervention research topics. COVID-19 includes early physiotherapy, a transition period, a home program, and rehabilitation. Figure 1 shows the Prisma flow chart of the available published literature on physical therapy for multiple sclerosis.

Result

The database search found a total of 132 results. Each stage of the research selection phase is depicted by a PRISMA flow diagram. Online Database Search used were Scopus, Embase, Medline, CINAHL, Pedro, PubMed, and Google Scholar. First-level screening for relevance was performed using titles, abstracts, and keywords. Of which, the non-relevant articles excluded were 47, and the duplicates that were excluded were 32. Six articles were excluded because of non-English text, and for two articles no full text was available. Full-text copies of publications were used for a second stage of screening for relevance. After the full texts were assessed using the inclusion/exclusion criteria, 27 studies and 18 conflicts were identified. Twenty-seven data needed to be assessed for eligibility after the researchers assembled and discussed each contradicting paper. Charting of data was performed by two independent authors. A sum of 16 RCTs was involved in this article. One cohort study was included. Four studies were based on review articles. In total, 1461 individuals with Multiple Sclerosis were involved in this review. They were involved despite the limits of specific publication forms because the scope of the review was to include all primary studies in the fields of physical therapy for multiple sclerosis, and the research question was to cover all primary research in the field of physical therapy for multiple sclerosis.

Early physiotherapy and transition care

Multiple sclerosis (MS) patients might appear with a variety of physical and psychological symptoms [9]. MS individuals remain to be less active than their general population counterparts. Many patients with MS are worried about their capacity to engage in physical activity [3]. All physiotherapy applications should be advised in such a way that they address the greatest amount of motor deficits possible. It is essential to avoid the immobilization of patients during acute illness owing to the effects of akinesia. Physiotherapy for these patients includes regular changes in body position to avoid bedsores, the use of passive exercises to avoid contractures, and the use of breathing exercises to minimize respiratory system complications. The prevention of urogenital infections and support with activities of daily life are critical components of patient treatment [7,10]. Early in the disease's progression, learning and memory problems might start to manifest. Cognitive impairment is thought to be a prognostic factor for the progression of the disease following the onset of Multiple Sclerosis. Memory and executive function impairment have a detrimental impact on the quality of life of patients and their basic activities of daily living. Identifying early executive dysfunctions may be beneficial in enrolling Multiple Sclerosis patients inappropriate rehabilitation interventions [11]. The rehabilitation program should take into account the disease's stage, degree of impairment, and neurological abnormalities. The therapy during remission is thus depending on the degree of disability and the treatment goals. The rehabilitation process should be continued with exercises that eliminate the problems created by the condition, and early rehabilitation should be considered [7]. Every patient with Multiple Sclerosis should have their healthcare professionals encourage and emphasize the effectiveness of physical activity as part of a healthy lifestyle. An early examination by a physical therapist familiar with Multiple Sclerosis is advised to develop a participant’s fitness and lifestyle physical exercise plan [3]. Although hospital-based rehabilitation programs appear to have a higher impact, numerous studies have found that home-based rehabilitation is helpful. Studies have shown that hospital-based patients recover significantly, whereas home-based patients suffer considerably, indicating the need for proper planning transition care rehabilitation [12].

Different physiotherapy approaches

Among the most incapacitating symptoms of MS is fatigue. Fatigue tends to affect more than two-thirds of multiple sclerosis patients and harms their ability to participate in a variety of everyday activities, their quality of life, and their ability to work [13]. Inpatient energy management education increased self-efficacy in energy-saving techniques and enhanced physical functioning, both of which are important aspects of quality of life [14]. Activity pacing is a method of breaking down one's everyday activities into smaller, more manageable segments that don't worsen symptoms, allowing for gradual improvements in activity. Activity pacing that is customized to physical activity behavior and tiredness enhances levels of physical activity without aggravating fatigue symptoms [15,16]. Multiple sclerosis patients have a significant prevalence of sleep problems. Exercise is a non-pharmaceutical, low-cost, and safe way for persons with MS to enhance their sleep quality. One of the physiologic processes causing these changes could be related to the rise in serotonin levels because of aerobic exercise [17]. Exercise can boost neurotrophic synthesis and secretion, although disability status has no impact on it [18]. Motor deficits are common in MS patients, which can lead to postural imbalance and walking problems. Patients with MS often benefit from high-frequency physical therapy to facilitate their postural balance and mobility [19]. Balance and hand dexterity can be improved with Halliwick and Aquatic Plyometric Exercise. The Aquatic Plyometric Exercise was demonstrated to be safe and effective in improving trunk control and dexterity [20]. In women with MS, aquatic exercise training increased functional ability, balance, and fatigued perceptions [13]. Balance and Eye-Movement Exercises for People with Multiple Sclerosis enhanced a variety of outcomes irrespective of whether or not brainstem/cerebellar lesions were evident, indicating that BEEMS can be used by ambulatory MS individuals who have at the very least impeded balance and fatigue [21]. Several MS patients with Virtual-Reality based and traditional balancing exercises improved their balance and mobility. VR-based training was found to be more effective in improving cognitive-motor performance and reducing falls than traditional training, whereas traditional exercises resulted in improved directional control [22,23]. In comparison to no exercise, the CoDuSe exercise enhanced balance and reduced perceived walking restrictions. The intervention decreased the number of falls and near-falls [24]. Various exercise techniques are widely used to treat ataxic symptoms in MS patients. Lumbar stabilization exercises or task-oriented training should be used as a supplement to enhance coordination and balance in individuals with ataxic multiple sclerosis [25]. An additional pain treatment method is a low-intensity upper-limb and breathing exercise program for the possible decrease of pain and enhancement of functional independence in both ambulatory and non-ambulatory MS patients [26]. When compared to MS controls, the Combined Training Program improves their balance, rate of force generation, and static strength muscles. CTP may help MS patients perform better in everyday tasks, including walking, sitting, and standing [4]. Patients with MS tend to have muscular strength and endurance impairments, notably in respiratory muscles, which decrease functional performance and physical capacity. Respiratory muscle weakness can also result in decreased lung function, which can lead to (aspiration) pneumonia or acute ventilatory failure, both of which are significant causes of death in MS patients [27]. Resistive inspiratory muscle training using a resistive threshold device is moderately effective postintervention for improving predicted maximum inspiratory pressure in people with mild to severe MS [27]. Ambulatory, home-based, and inpatient rehabilitation settings should all be included in rehabilitation program settings [2].

Home program

Calisthenic exercises are used in a variety of rehabilitation regimens, and they have been shown to improve fatigue, muscular strength, and balance as well as a standard neurorehabilitation program [12]. For patients with MS, mobility is a key concern. After diagnosis, around 80% of patients will have reduced activity within 10-15 years. After a while, about 25% of patients are reliant on wheelchairs [28]. Mobility includes more than walking; it also includes standing, transferring, and getting in and out of bed. Patients with multiple sclerosis consume a lot of time sitting; usually, the ability to shift positions is hampered. Generally, interventions have been resource-demanding, requiring regular monitored sessions with a physical therapist or sports therapist in outpatient or clinical settings, as well as the use of costly equipment that cannot be utilized at home [28]. The value of healthcare provision has been found to rise significantly when condition severity/immobility rises [29]. People with significant physical limitations ensure efficient self-management approaches that are cost-effective and feasible to apply to maximize their physical activity participation [28]. Health and social care policy emphasizes the requirement to enable individuals to self-manage through cooperation and self-management programming with a focus on a forthcoming National Health Service that includes community-based programs that encourage self-care and lifestyle behavioral modification [29]. The one alternative is regularly assisted standing with an assisted device, such as a standing frame, that may be utilized in an individual’s home. Patients with limited movement, coordination, or lower limb or trunk control can use standing frames to expend time in supported standing. Standing has been shown to improve antigravity muscular strength, provide extended weight-bearing muscle stretch, improve cardiopulmonary efficiency and preserve bone density [28]. Tele-exercise is a potential form of research that might benefit patients with MS significantly. Offering various kinds of training in the convenience of an individual's own home will promote regularity and continuity in programs that are constrained by travel distance, fees, the need for a driver, absence from work, or being the main caregiver [30]. Similarly, while most MS and primary healthcare institutions are unable to provide entire rehabilitation programs in clinics, remote training may be a viable option for delivering these crucial skills to patients who are unable to access such sessions for several reasons [30]. More than half of all multiple sclerosis patients suffer from cognitive impairment. For enhancing cognitive functioning in patients with MS, 12 weeks of training with an adaptive cognitive remediation training session outperformed an active control of playing regular computer games. One significant advantage of such rehabilitation is that the intervention could be accessed from the comfort of one's own home [31]. The evidence-based complementary and alternative medicine exercise program, which included pilates, yoga, and neurological rehabilitation exercises like Spine Twist, Child's Pose, and Multidirectional Reaching with Upper Extremities and/or Lower Extremities with Cognitive Component (Conversation), was effective. Telerehabilitation offers the potential to address important service gaps for MS patients who have little or no access to therapeutic exercise/rehabilitation programs [30].

Rehabilitation of MS in COVID-19 pandemic

The constraint imposed by COVID-19 affects individuals with multiple sclerosis [28]. People with MS would be particularly affected since they might be considered an at-risk population group for severe COVID-19 in several ways, and the extent of data needed to treat MS has increased significantly. This meant a greater risk of transmission in this group of patients among the frailest population, especially for moderately disabled patients with MS who had been enrolled in their rehabilitation. In some situations, the widespread impact of COVID-19 made it difficult to maintain the prior level of care [32,33]. Individuals who had mental health problems before confinement and social isolation may be more vulnerable to unfavorable psychological repercussions [34]. There is an increased need for health professionals in MS to truly comprehend the situation and possess the necessary information and abilities in self-management of physical activity and mental health issues [8]. To help manage the psychosocial effects of COVID-19, psychology services can be provided by contacting them via telecommunication and following up with them regularly. Teleassessment and rehabilitation via individual or group videoconferences, as well as supplying patients with specific online resources during follow-up conversations, might be an option [32]. MS patients should be counseled not to modify their MS therapy without first consulting their neurologist. Individuals with MS should follow World Health Organization (WHO) and regional or national health authority guidelines on preventative measures to reduce COVID-19 transmission in the general community. Only a few of these include respiratory hygiene, frequent handwashing with an alcohol-based hand rub or soap, and social isolation [35].

Discussion

The research included in this study was of varying quality. There were a few studies that were of poor quality, but there were also some that were well-designed and of excellent quality. Physical therapy research for patients with MS has increased in terms of both quality and quantity.

This scoping review examines the literature on Multiple Sclerosis, including evidence of early physiotherapy for MS patients, difficulties for patients and their relatives during the transition phase of patients from hospital to home, what care should be given during this phase, what home program should be given to these patients, and different physiotherapy approaches being used in MS patients and observing the current COVID epidemic situation, rehabilitation of these people in the face of adversity.

Some studies show the importance of early physiotherapy for Multiple Sclerosis patients in preventing the complications in long run. Studies have shown that hospital-based patients recover, whereas home-based patients suffer considerably, indicating the need for proper planning transition care rehabilitation and the need for telerehabilitation amid the COVID-19 pandemic so that no patient should be left without rehabilitation and no further complications should develop, therefore there is an increased need for health professionals in MS to truly comprehend the situation and possess the necessary information and abilities in self-management of physical activity and mental health issues [7,30].

Elisabet Guillamó et al. found that supervised training combined with a home program was safe and feasible in their pilot study of 40-week structured physiotherapy treatment for adults with Relapsing-Remitting Multiple Sclerosis [1]. Nadine Akbar et al. conducted a study on the effects of Progressive resistance exercise training on individuals with MS and severe fatigue. They presented preliminary evidence supporting the caudate as a potential neural substrate for Progressive resistance exercise training's positive effects on fatigue in MS patients [13].

A study was conducted by Freeman et al. in which individuals with multiple sclerosis were assigned at random to receive regular therapy only or regular therapy plus a standing frame training. And they concluded that frequent usage of the standing frame together with regular therapy results in noticeable benefits in motor performance [28].

In a randomized control trial conducted by Gunn H et al. on progressive MS patients, they were given regular therapy with Balance Right in MS (BRiMS) or regular therapy only. The conclusion says that trial protocols are practicable and suitable, and even the result participation rate met the prior progression requirements [29].

## Conclusions

There are few articles about transition care of multiple sclerosis patients when they are transferred from hospital to home, how difficult it is for them and their family members to learn patient care techniques, and how there is a paucity of knowledge on the disease's condition and prognosis, as the existing articles in this scoping review show.

In addition, there are few papers regarding the rehabilitation of MS patients in the COVID-19 epidemic, as well as the utilization of telerehabilitation to solve this issue.
